# Designers Drugs—A New Challenge to Emergency Departments—An Observational Study in Poland

**DOI:** 10.3390/medicina56070354

**Published:** 2020-07-17

**Authors:** Rakesh Jalali, Paula Dmochowska, Izabela Godlewska, Justyna Balmas, Katarzyna Młynarska, Krzysztof Narkun, Andrzej Zawadzki, Marcin Wojnar

**Affiliations:** 1 Emergency Medicine Department, School of Medicine, Collegium Medicum, University of Warmia and Mazury in Olsztyn, ul. Żołnierska 18 10-561 Olsztyn, Poland; paula.dmochowska@gmail.com (P.D.); iza.gdlwsk@gmail.com (I.G.); balmas.j@gmail.com (J.B.); karolina.walczak@uwm.edu.pl (A.Z.); 2 Clinical Emergency Department of Regional Specialist Hospital in Olsztyn, Żołnierska 18, 10-561 Olsztyn, Poland; 3 Pediatric Emergency Department of the Provincial Specialist Children′s Hospital in Olsztyn, Żołnierska 18a, 10-561 Olsztyn, Poland; kajmar1@interia.pl (K.M.); kn.med@wp.pl (K.N.); 4 Department of Psychiatry, Medical University of Warsaw, Nowowiejska 27, 00-665 Warsaw, Poland; marcin@wum.edu.pl

**Keywords:** new psychoactive substances, designer drugs, pediatric emergency department, emergency medicine department

## Abstract

*Background and Objective*: In the last decade, the phenomenon of using new psychoactive substances (NPS), called designer drugs, has been on rise. Though their production and marketing in Poland is prohibited, reports of the Supreme Audit Office noted that young people are increasingly reaching for new intoxication agents in the form of designer drugs. There is a significant increase in the number of patients with NPS abuse admitted to the emergency departments. As NPS cannot be detected by standard tests for the presence of psychoactive substances, it is difficult to choose the appropriate therapeutic intervention. Therefore, the aim of the present study was to evaluate the patient characteristics in the population of adults and children suspected of using NPS and formulate the protocol for diagnosis and treatment. *Materials and Method*: The paper is based on a retrospective analysis of medical records of hospitalized patients in the Clinical Emergency Department of The Regional Specialist Hospital in Olsztyn (SKOR WSS, emergency department (ED)) and the Pediatric Emergency Department of the Provincial Specialist Children′s Hospital in Olsztyn (SORD WSSD, pediatric emergency department (PED)) between years 2013 to 2018. The patient records related to their general symptoms at admission, mental state and laboratory diagnostic tests were evaluated. *Results*: The majority of patients hospitalized due to the suspected use of NPS were adolescents in 2013–2016 and a reversal of this trend was observed in 2017–2018 when number of adults admitted to the emergency department (ED) due to NPS use was higher. The NPS abuse was significantly higher among male patients, alcoholics, people using other psychoactive substances, patients suffering from mental disorders and teenagers in difficult socio-economic family situations. Whereas, the most common symptoms among pediatric patients were co-ordination disorder and aggression, in adults mainly tachycardia and aggression was observed. The laboratory tests in significant number of adult patients showed leukocytosis and ketonuria. *Conclusions*: In the present study, no unambiguous toxidrome or biochemical pattern characteristic for using NPS was observed. However, evaluation of blood morphology, coagulation parameters, liver and kidney function can be helpful in the diagnostic and therapeutic process. Symptomatic treatment of patients, fluid therapy and sedation was sufficient in most cases to resolve the patient symptoms in 48 h.

## 1. Introduction

In the last decade, the phenomenon of using new psychoactive substances (NPS), is on the rise and is posing a diagnostic and therapeutic challenge, especially for emergency physicians [1,2,3]. NPS, commonly called “legal highs”, are psychoactive substances not included in the list of drugs and psychotropic substances annexed to the Act of 29 July 2005 on counteracting drug addiction. In the latest version of the same Act of 6 August 2018, the concept of “new psychoactive substances” is included and defined as prohibited substances [4]. According to the definition of the World Health Organization (WHO), legal highs are divided into nine main groups, including synthetic cannabinoids, synthetic cathinones and hallucinogens. The NPS acts by mechanisms similar to the known drug classes. However, the effects of their action maybe unpredictable [5], and the observed symptoms tend to be more severe than those caused by classic drugs [6]. This could be due to their chemical structure and greater affinity for analogous receptors, for example, synthetic cannabinoids usually work as would be expected from their affinity to CB1 receptors but during research it has been found that halogenation of the compounds has led to the development of new analogues with higher potency. Baumeister et al. used the example of AM-220, which has 10 times stronger binding to the CB1 receptor than its precursor JWH-018 [7,8]. However we cannot exclude other reasons for observing greater toxicity in the population, for example, because of substance impurities or other unknown receptors. In addition, NPS are characterized by high content of individual components (such as drug for sedation, sympathomimetic drugs or anticonvulsants) and variable qualitative and quantitative compositions [1,9].

The production and sale of legal highs in Poland is prohibited. However, the reports of the Supreme Audit Office highlight the growing pathological phenomenon among young people, who are increasingly choosing NPS as a substitute for narcotics [10]. The first reports about legal highs appeared in the 1960s. NPS dealers have been already looking for a way to circumvent drug law calling legal highs ′research substances′, ′incense′, ′washing salts′ and ′vegetable food′, with labels on the packaging showing the possible content of hazardous substances or information that ′the product is not suitable for human consumption′ and prohibiting their sale to minors [11]. This NPS sales tactic has been functioning in Poland until today.

The popularization of the use of NPS and their easy availability results in increasing number of intoxicated patients and admissions to the ED. These substances are usually impossible to be detected by the standard tests for the presence of drugs, which makes it difficult to take appropriate therapeutic steps. This is particularly important in the case of acute poisoning [12].

The NPS use has been associated with violence and accidents, health complications leading to death and neuropsychiatric symptoms [13]. In addition to the side effects caused by the use of individual psychoactive substances, the mixed composition of NPS can lead to unpredictable harmful effects, such as damaged liver, heart toxicity, kidney and respiratory problems [14].

The aim of the study was to assess the use of NPS in the population of adults and children in Olsztyn and the surrounding area based on the cases of patients hospitalized in two reference regional hospitals. The symptoms after NPS consumption, the connection between the patients′ mental states and the use of psychoactive substances, and the prevalence of NPS dependence were assessed. The other aim of the study was to assess the diagnosis and emergency therapeutic management in patients with NPS abuse.

## 2. Materials and Methods

The present observational study, approved by the local ethical committee (document number WL-KB.0020.0173.71.2020), was based on a retrospective analysis of medical records of patients hospitalized in the Clinical Emergency Department of The Regional Specialist Hospital in Olsztyn (SKOR WSS; emergency department, ED) and the Pediatric Emergency Department of the Provincial Specialist Children’s Hospital in Olsztyn (SORD WSSD; pediatric emergency department (PED)) between years 2013 and 2018. The patient files were provided to researchers with names and surnames being substituted with the codes and all the data collection methods were in compliance with the Helsinki Declaration.

A group of people categorized as suspected of NPS use was selected from all patients by analyzing the history taken from the patient, from their family or bystanders who called the ambulance. In the patient evaluation, we primarily included general symptoms, mental state and laboratory test results. The patients were evaluated based on Glasgow coma score (GCS), Pediatric Early Warning Score (PEWS) and National Early Warning Score (NEWS), with the following validation values:

- GCS [15]: evaluation of eye, speech, and motor functions;
- NEWS [16]: seven routinely collected vital signs, including respiratory rate, oxygen saturation, temperature, blood pressure, pulse and AVPU response;
- PEWS [17]; three routinely collected vital signs including capillary refill, behavior and respiration rate.

General medical condition of a patient was considered good if GCS was above 13, NEWS in the range 0–1 and PEWS in the range 0–1. Patients were classified in moderate general condition if GCS, NEWS and PEWS were in the ranges of 11–13, 2 and 2, respectively, and in severe general condition if GCS was below 11, EWS was 4 or above and PEWS was greater than 3.

### Statistical Analysis

The proportions between subgroups were compared by the chi-square: the two-sided Fisher’s exact test. The difference in median of age between adults and minors was estimated by the Mann–Whitney test. The normality distribution of data was assessed by Shapiro–Wilk test. The significance level was set at *p* < 0.05. The analysis was conducted using TIBCO Software Inc. (2017), Statistica (data analysis software system), version 13 (http://statistica.io).

## 3. Results

**Figure 1:**
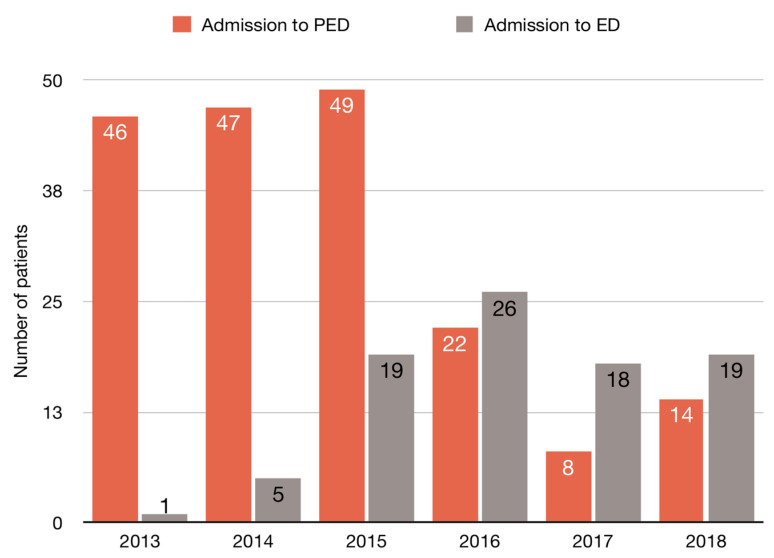
Number of admissions in ED (Emergency Department) and PED (Pediatric Emergency Department) in years 2013 to 2018.

**Figure 2:**
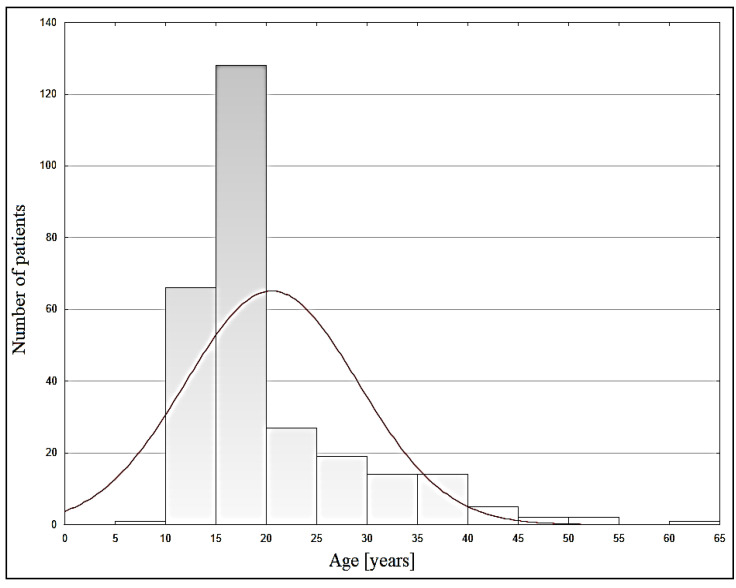
Age distribution in the study population.

We evaluated the number of hospitalizations among juvenile and adult patients due to the NPS use in the years between 2013 and 2018. The NPS use was found to be twice as high in juvenile patients as the number of hospitalizations among adults. The total number of hospitalizations was 186 (66% of all NPS use patients) in the Pediatric Emergency Department (PED) of the Provincial Specialist Children′s Hospital in Olsztyn and 96 (34%) in the Hospital Emergency Department WSS in Olsztyn (Figure 1). In the analysis, a total of 282 patients were included in the age range of 10–63 years: a median of 17 years (16–23 IQR (interquartile range)) (Figure 2). In both the analyzed groups, the majority of patients were male (ED: 90 males vs. 6 females; PED:149 males vs. 47 females). The characteristics of the study groups are presented in Table 1.

**Figure 3:**
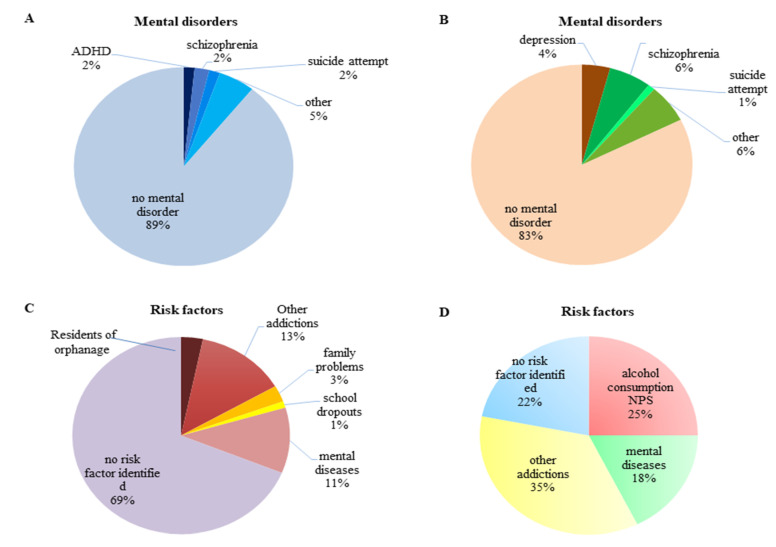
Pie charts depicting classification of mental disorders and risk factors associated with prevalence of NPS use in juveniles (**A**,**C**) and in adults (**B**,**D**). ADHD: attention deficit hyperactivity disorder. NPS: new psychoactive substances.

The median age among adults was 28 years (IQR: 23–36), and among minors it was 16 years (IQR: 15–17) (*p* < 0.001). An upward trend was observed in the number of adult patients suspected of NPS use admitted to the ED in the years 2013–2018. In contrast to ED, the number of hospitalizations of minors in PED in 2013–2015 remained stable, at about 50 admissions per year. Since 2016, adults have been more frequently hospitalized (26 vs. 22 in 2016) and the number of hospitalizations in PED due to taking NPS had decreased by more than a half. The trend in predominance of the number of patients hospitalized due to taking NPS in ED vs. PED was maintained in 2017 and 2018 (Figure 1). The patients with mental disorders and previous history of addictions were at greater risk of using NPS. The mental disorder classifications in juvenile and adult NPS use patients and other risk factors are presented in Figure 3.

### 3.1. General Condition and Symptoms

**Figure 4:**
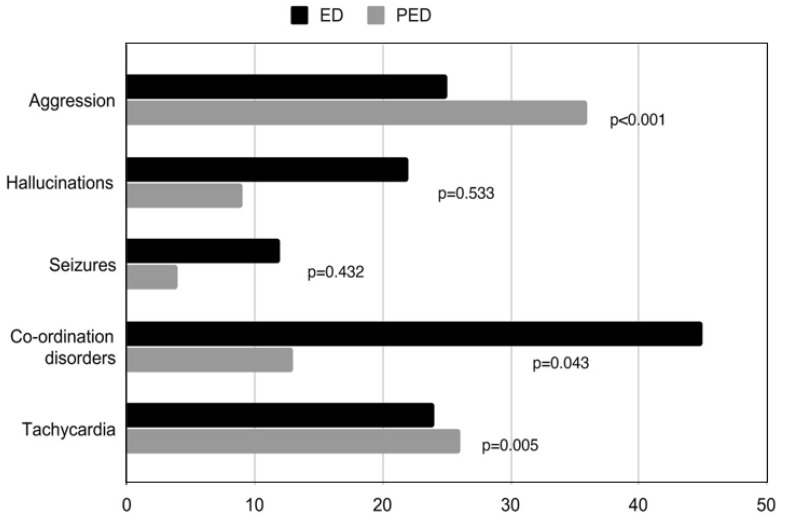
Comparison of the main symptoms of NPS use in adults (admitted to ED) and children (admitted to PED).

In both groups of hospitalized patients, similar signs and symptoms of NPS use were noted. Both somatic (i.e., brady- or tachycardia, vomiting, increased blood pressure, stress, co-ordination disturbance and dysarthria) and mental (e.g., behavioral deviations, including verbal and physical aggression, cheerfulness, positive symptoms, psychomotor retardation and consciousness disturbances) symptoms were observed (Figure 4). A total of 36 out of 96 patients in ED (37.5%) presented aggressive behavior, of which over one third required direct coercion. In the PED, only 25 out of 186 patients (13%) presented aggressive behavior.

Most pediatric patients were admitted to the PED in good general condition (54%); severe cases accounted for 6% of all admissions. The deviations from normal laboratory parameters were observed in 23% (*n* = 44) of pediatric patients and these included hypokalemia (6%), leukocytosis (4.3%), metabolic (2%) or respiratory acidosis (2%) and elevated creatinine (3%), CRP (3%) and LFTs (3%), we also observed ECG deviations, such as prolonged QTc and presence of PVCs. One of the patients was also diagnosed with enlarged liver and decreased echogenicity of kidneys. Similarly, in the adult group, nearly two thirds of patients on admission presented in good general condition (65%), the percentage of severe poisoning was noted in 4% of cases. The deviation from laboratory parameters was observed in 27% (*n* = 26) cases that included hypokalemia (2%), hypernatremia (2%), leukocytosis (18%), respiratory acidosis (1%), ketonuria and elevated creatinine (4%). A medical state was evaluated by the first contact with the patient paying attention to the cardiovascular, respiratory and nervous system performance. Scales, such as GCS, PEWS or NEWS, were used to evaluate the general condition of a patient. In both groups of patients, symptomatic treatment lasting from several hours to about a day ensured the resolution of mental and somatic symptoms, and ~ 90% of patients were discharged home in a good general condition.

The hospitalization time did not usually exceed 24 h. However, we noticed that in the pediatric group of patients, there were four cases admitted in severe condition who stayed in PED for more than 24 h, of which two cases were admitted to ICU for 7–8 days and then transferred to the psychiatric ward. All adult and pediatric patients were divided into three groups: hospitalized in 2013–2014, 2015–2016 and 2017–2018. We noticed an increase in the median age of patients over the years, a significant increase was observed in number of patients presenting with an aggressive attitude, and in the anamnesis we noted more mental illnesses. Over one fifth of hospitalized patients required further hospitalization in the provincial psychiatric treatment center (WZLP). The data are presented in Table 2.

### 3.2. NPS and Other Drugs

In both the study groups, approximately 30% of patients had previous admissions associated with taking NPS. 35% of ED patients hospitalized because of taking NPS declared their dependence on other psychoactive substances. The most frequently used were THC (tetrahydrocannabinol), amphetamine, GBL (gamma-butyrolactone, a component of rim cleaner fluid), alcohol and other drugs. In the pediatric population, other addictions were noted in 22% of hospitalizations: THC and alcohol were the most commonly used among minors. Combining alcohol with NPS occurred in both study groups (minors 10% vs. adults 25%).

### 3.3. NPS and Mental Disorders

Among patients hospitalized in PED, in 11% of cases there was a history of organic disorders, ADHD (attention deficit hyperactivity disorder) or schizophrenia. Three hospitalized patients had attempted suicide in the past. In ED, 18% of patients had a mental illness, mainly schizophrenia and depression. Due to the worsening of the disease and aggressive behavior, some of these hospitalizations required the police assistance and further treatment of the patient at the Provincial Psychiatric Treatment Center in Olsztyn (19 adults and 8 minors).

### 3.4. Risk Factors

Based on the information collected, additional risk factors for the intake of NPS were identified. One of the most important was the social and family situation. Six juvenile patients under the supervision of a probation officer or permanently residing in an orphanage were found in the study group. Nearly one fifth of hospitalization of juvenile patients included children with educational problems, conflict with parents, frequent fights or consuming additional psychoactive substances.

The analysis also showed that in the period of study, NPS consumption most often occurred in the months from April to June and from September to October. In the adult population, nearly a third of patients after consuming NPS were hospitalized in December 2016.

### 3.5. Additional Medical Tests

In the years 2013–2016, 78% of hospitalizations in PED and 90% in ED did not reveal any deviations in the laboratory tests. An increase in the number of deviations in laboratory tests was observed in the years 2017–2018, when abnormalities were noted in 73% of adult patients. One of the symptoms observed was dehydration manifested, inter alia, in electrolyte disorders, elevated creatinine level and ketonuria. The recurring abnormality was leukocytosis, without any symptomatic infection, which in adult patients was associated with repeated hospitalizations due to the intake of NPS.

In seven patients, superficial skin injuries or infections were seen in the physical examination. In seven cases, respiratory acidosis was observed. Six of these patients reported prior use of NPS in the form of a cigarette or e-cigarette. For these patients, there were no deviations during examination as respiratory rate, and the oxygen saturation level was normal.

One of the pediatric patients showed the evidence of liver damage (elevated AST 254 U/L, LDH 316 U/L and INR 1.48). In addition, dehydration and inflammation were observed and the patient was admitted in a state of metabolic acidosis. He was transferred to the intensive care unit because of his general condition associated with multiple seizures unresponsive to diazepam. The patient was addicted to many psychoactive substances, but their presence and alcohol were not detected in laboratory tests.

Individual cases requiring additional intervention have also been reported. One of the patients was referred to the Pomeranian Center of Toxicology due to three times exceeding the therapeutic norm of carbamazepine and the history of simultaneous consumption of NPS. One patient required a gynecological consultation due to being the victim of an attempted rape while being under the influence of psychoactive substances. The patient′s blood had 2.17 g/L of alcohol and tests for psychoactive substances were negative, but the patient′s behavior was inadequate, justifying the doctors′ suspicion of taking NPS.

### 3.6. Other Health Threats

In the population we observed, the presence of injury under the influence of NPS was noted in 10 adult patients and 21 children; most often the injuries that included mainly abrasions, bruises and cut wounds, were caused as a result of fights. In patients with head injuries associated with taking NPS (usually as a result of fall from standing height), we did not notice any post-traumatic changes in imaging tests.

Among the risk behaviors we observed driving under the influence of NPS (two men were brought to ED assisted by the police after a road check due to dangerous driving).

## 4. Discussion

The phenomenon of using NPS is currently extensively described in the literature and the main thrust is to identify chemical compounds contained in legal highs, to analyze the symptoms of poisoning observed in ED [14,18], risk groups [19] and the effectiveness of a treatment [20]. The NPS constituents are one of the most difficult chemical intoxications to diagnose [21]. However, this retrospective study presents analysis of juvenile and adult patients suspected of NPS uses classified on the basis of ICD-10 as no specific laboratory tests were available to detect chemical constituents of NPS. The data was collected from two main centers in Olsztyn: SKOR WSS (ED), where the annual number of admissions reaches 28,000 and SOR Children′s Hospital (PED) admitting nearly 10,000 patients under 18 years old per year. The analysis of patient data included in the study from two centers made it possible to assess the use of NPS and its effects in the Warmia and Mazury region in both age groups.

The lower incidence of NPS intake in adults that occurred at the beginning could have been caused by the lack of unequivocal symptoms of taking NPS, as well as lower reporting in adult patients to ED due to the lack of supervision of relatives and lower public vigilance towards adults. It seems that the growing awareness of doctors in this matter results in increased diagnostic vigilance, and thus, greater recognition of NPS poisoning in the adult population. On the other hand, a lower number of pediatric admissions in 2016–2018 could be related to the changing laws in Poland that resulted in better control over the sale of such substances to the minors.

Henshall et al. [22] in 2015 reported that the average age of hospitalized patients using NPS was 33 years; the youngest was 17 and the oldest 57. Most of the patients were male. The paper published a year earlier by the Crime and Policing Analysis Unit [23] showed that the average age of people using NPS was 24 years. Using the international database in 2016, the Global Drug Survey [24] determined this age to be 28 years. There are currently no studies analyzing larger groups of minors to which the results of this study could be compared. The youngest patient in our study was 10 and the oldest 63 years old. The majority were male patients, which is consistent with the results of other Polish researchers [25]. This type of trend is also observed worldwide. Kamijo et al. [13] reported that in the Japanese population predominantly, NPS users are males at the age of 30. In the face of the growing number of people admitted to the ED with suspected consumption of psychoactive substances and alcohol, it is important to quickly assess the general condition of the patient, which affects further diagnostic and therapeutic activities. The general condition of examined patients at the admission was similar to the studies conducted by Sawicka et al. [26]. The most frequent clinical symptoms observed were anxiety, agitation and vertigo. Average pulse rate was within normal limits, however authors noted slight tendency towards tachycardia [26]. In the adult group we studied, patient characteristics include aggression and sinus tachycardia, and among juvenile population mainly co-ordination disorders and aggression were noted.

Analysis of the symptoms presented by patients and taking into account the type of suspected ingested substance, we were not able to isolate corresponding toxidrome that would describe the pattern of behavior and symptoms characteristic of consumed groups of NPS. In the described study group, it was possible to obtain a good response to symptomatic treatment that included fluid therapy and supplementation with specific electrolytes in dyselectrolytemia, controlling diuresis in case of abnormal renal function and use of sedatives if patient was agitated. However, one should definitely pay attention to the unpredictability of the effects of NPS, possible complications of their use and potential mutual intensification of the effects of the substance in case of taking several different psychoactive substances, that could cause the inability to reverse their effect by means of a specific antidote [27].

In recent years, increasing number of new compounds are classified as NPS [24]. The authors investigating this phenomenon emphasize that the main factors contributing to the increasing interest in products from the group of NPS are ease of access and low price compared to traditional drugs [28,29]. Currently, synthetic cannabinoids are the most frequently chosen NPS in the world [30]. In Poland, synthetic cathinones, e.g., mephedrone, are the most popular [25]. The use of synthetic cannabinoids may cause severe symptoms compared to natural cannabinoids [31,32,33]. The study by Kassai and co-workers [28] pointed to the possible consequences of chronic use: men surveyed reported the unpredictable effects of each subsequent next use, as well as a rapid change in the overall experience. Initially, they described them as positive, with a tendency of rapid progression towards negative feelings accompanying the sense of addiction. Patients felt isolated and had positive symptoms over time. According to some studies, chronic use of synthetic cannabinoids may lead to the onset of depressive symptoms [34]. The above observations also confirm the results of this study, both among adults and minors.

NPS, like other psychoactive substances, carry the risk of dangerous sexual behavior. They can also be used for rape by potential perpetrators. There is an added risk of contracting sexually transmitted diseases, including HCV or HIV [35]. In addition, taking NPS predisposes the occurrence of injuries [35], for instance dangerous craniocerebral injuries [36]. Preliminary studies in mice [37] showed that chronically used synthetic cannabinoids can affect the cerebellar function, which in turn leads to motor disorders.

The research presented in this paper shows that taking NPS can also lead to life-threatening situations. The case of a juvenile patient presenting with status epilepticus that we cited requires hospitalization in the intensive care unit ICU. Byard et al. [36] described a case in which a 19-year-old boy suffered a lethal craniofacial injury as a result of an untreated epileptic seizure. A number of compounds classified as NPS were posthumously detected in this patient. The number of cases with unpredictable consequences for users will probably rise with the increasing number of synthesized derivatives of new psychoactive substances.

## 5. Limitations

The major limitation of this study was the unavailability of a detailed information about subclasses of NPS or other substances used by patients. The information in patient charts revealed NPS types of which patients had declared their use at the time of admission or were found by the patient’s side by the para-medical staff. Most of these substances had local Polish names and it was impossible to find their composition except for that of “express cocktail” that was noted in the history of some of the patients and is composed of clonazepam 4 mg and ketoprofen 200 mg. As the data was collected retrospectively we could not record further details that could have been useful to identify the type of NPS used. The problem was compounded by the unavailability of blood or urine tests specific for detecting NPS components. Moreover, we used data compiled by other doctors or nurses that could lead to missing some symptoms or an incomplete/inappropriate recall by the patients. Another limitation was the way the patients were coded according to ICD-10. If they were described drug users, only then were they included as NPS users. It was possible that some cases could not be properly described by 10th revision of the International Statistical Classification of Diseases and Related Health Problems (ICD- 10), which made it impossible to find every NPS user who visited the emergency department.

## 6. Summary

**Figure 5:**
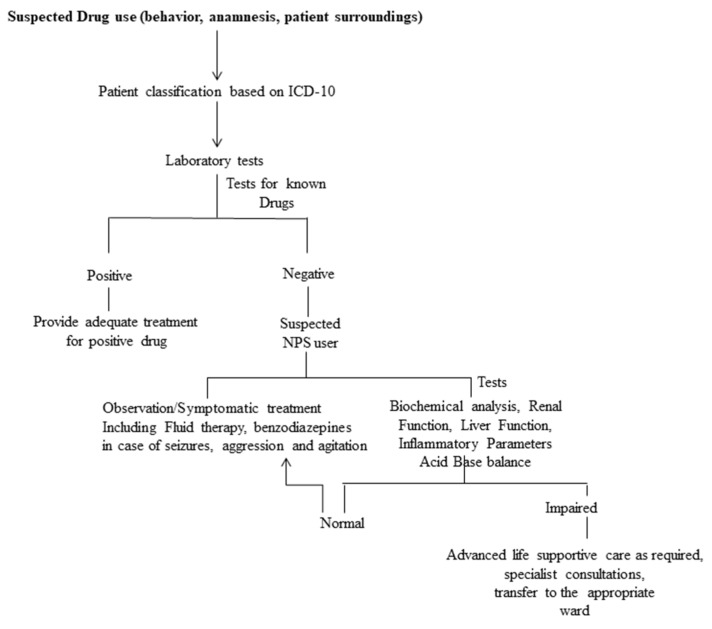
A general strategy for the management of patients suspected of NPS use.

Greater predisposition to taking so-called “legal highs” were shown by male patients in both groups, alcoholics and patients using other psychoactive substances, patients with mental disorders (mainly schizophrenia and depression) and young people in difficult socio-economic and family situations. There was no unambiguous toxidrome or biochemical pattern that was characteristic of taking these substances. According to the treatment protocols used in our hospitals, a general therapeutic strategy as shown in Figure 5 should be primarily guided by the clinical picture. The results of basic laboratory tests, including creatinine, liver function tests (LFTs), leukocytosis or blood counts could allow detection and monitoring of progression of patient’s general condition. Monitoring vital functions and fluid therapy was helpful during treatment. Moreover, evaluation of blood morphology, coagulation parameters, liver and kidney function can be helpful in the diagnostic and therapeutic process, which in our study was based mainly on symptomatic management. The symptomatic treatment was effective and sufficient in most patients.

## Figures and Tables

**Table 1:** Patient characteristics of study groups.

	All		PED		ED		*p*
All *n* (%)	282	(100)	186	(66)	96	(34)	
Sex *n* (%)							
female	53	(19)	47	(25)	6	(6)	<0.001
male	229	(81)	139	(75)	90	(94)	
Age [years] (median, IQR)	279	17 (16–23)	16 (15–17)		28 (23–36)		<0.001
no data	3				3		
Risk factors							
Mental disorders *n* (%)	37	(13)	20	(11)	17	(18)	0.135
Other diseases *n* (%)	19	(7)	5	(3)	14	(15)	<0.001
Other addictions *n* (%)	74	(26)	40	(22)	34	(35)	0.015
Addictions							
THC *n* (%)	9	(3)	6	(3)	3	(3)	0.964
Amfetamin *n* (%)	5	(2)	2	(1)	3	(3)	0.217
GBL *n* (%)	6	(2)	2	(1)	4	(4)	0.088
Alcohol *n* (%)	20	(7)	7	(4)	13	(14)	0.002
Main symptoms of NPS							
Aggression *n* (%)	61	(22)	25	(13)	36	(37.5)	<0.001
Hallucinations	31	(11)	22	(12)	9	(9)	0.533
Seizures	16	(6)	12	(6)	4	(4)	0.432
Co-ordination disorders *n* (%)	58	(21)	45	(24)	13	(14)	0.043
Tachycardia *n* (%)	50	(18)	24	(13)	26	(27)	0.005
During hospitalization:							
Positive test of alcohol *n* (%)	43	(15)	19	(10)	24	(25)	0.002
The need for hospitalization in WZLP *n* (%)	27	(10)	8	(4)	19	(20)	<0.001
Earlier hospitalizations due to NPS *n* (%)	84	(30)	54	(29)	30	(31)	0.78
Transferred to another department/consulted *n* (%)	29	(10)	13	(7)	16	(17)	0.021
Medical Condition *n* (%)							
good	163	(58)	101	(54)	62	(65)	0.25
moderate	104	(37)	74	(40)	30	(31)	
severe	15	(5)	11	(6)	4	(4)	

WZLP: provincial psychiatric treatment center. IQR: interquartile range. PED: pediatric emergency department. ED: emergency department. THC: tetrahydrocannabinol. GBL: gamma-butyrolactone. NPS: new psychoactive substances.

**Table 2:** Variability in characteristics of all hospitalized patients, pediatrics and adults, over the years 2013–2018.

	2013–2014		2015–2016		2017–2018		*p*
All *n* (%)	99	(35)	116	(41)	67	(24)	
Sex *n* (%)							
female	22	(22)	24	(21)	7	(10)	0.129
male	77	(78)	92	(79)	60	(90)	
Age [years] (median, IQR)	16 (14–17)		17 (14–36)		23 (15–39)		<0.001
no data	3		2		1		
Risk factors							
Mental diseases *n* (%)	4	(4)	19	(16)	14	(21)	0.003 ^§^
Other diseases *n* (%)	1	(1)	9	(8)	9	(13)	0.006 ^†^
Other addictions *n* (%)	21	(21)	35	(30)	18	(27)	0.327
Addictions							
THC *n* (%)	3	(3)	3	(3)	3	(4)	0.777
Amfetamin *n* (%)	1	(1)	0	(0)	4	(6)	0.010 ^§§^
GBL *n* (%)	2	(2)	4	(3)	0	(0)	0.296
Alcohol *n* (%)	2	(2)	8	(7)	10	(15)	0.006 **
Main symptoms of NPS							
Aggression *n* (%)	6	(6)	32	(28)	23	(34)	<0.001 ^¥^
Hallucinations	11	(11)	8	(7)	12	(18)	0.072
Seizures	5	(5)	7	(6)	4	(6)	0.946
Co-ordination disorders *n* (%)	24	(24)	17	(15)	17	(25)	0.119
Tachycardia *n* (%)	12	(12)	21	(18)	17	(25)	0.089 ^^
During hospitalization:							
Positive test of alcohol *n* (%)	9	(9)	16	(14)	18	(27)	0.006 *
The need for hospitalization in WZLP *n* (%)	5	(5)	8	(7)	14	(21)	0.001 ^$^
Earlier hospitalizations due to NPS *n* (%)	25	(25)	38	(33)	21	(31)	0.463
Transferred to another department/consulted *n* (%)	7	(7)	14	(12)	8	(12)	0.426
Medical Condition *n* (%)							
good	58	(59)	52	(45)	53	(79)	<0.001 ^
moderate	36	(36)	54	(47)	14	(21)	
severe	5	(5)	10	(8)	0	(0)	

* significant differences between subgroups 2015–2016 vs. 2017–2018 and 2013–2014 vs. 2017–2018. ^§^ significant differences between subgroups 2013–2014 vs. 2015–2016 and 2013–2014 vs. 2017–2018. ^†^ significant differences between subgroups 2013–2014 vs. 2015–2016 and 2013–2014 vs. 2017–2018. ^¥^ significant differences between subgroups 2013–2014 vs. 2015–2016 and 2013–2014 vs. 2017–2018. ^ significant differences between subgroups 2013–2014 vs. 2017–2018 and 2015–2016 vs. 2017–2018. ^^ significant difference between subgroups 2013–2014 vs. 2017–2018. ^$^ significant differences between subgroups 2013–2014 vs. 2017–2018 and 2015–2016 vs. 2017–2018. WZLP: provincial psychiatric treatment center. IQR: interquartile range. PED: pediatric emergency department. ED: emergency department. ** significant difference between subgroups 2013–2014 vs. 2017–2018. ^§§^ Significant difference between subgroups 2015–2016 vs. 2017–2018.
